# Mutational Frequencies of SARS-CoV-2 Genome during the Beginning Months of the Outbreak in USA

**DOI:** 10.3390/pathogens9070565

**Published:** 2020-07-13

**Authors:** Neha Kaushal, Yogita Gupta, Mehendi Goyal, Svetlana F. Khaiboullina, Manoj Baranwal, Subhash C. Verma

**Affiliations:** 1 Department of Biotechnology, Thapar Institute of Engineering and Technology, Patiala 147004, India; kneha0563.nk@gmail.com (N.K.); yogita.gt@gmail.com (Y.G.); goyalmehendi700@gmail.com (M.G.); 2 Talwar and Talwar Consultants, Mohali 160055, India; 3 Department of Microbiology and Immunology, University of Nevada, Reno, NV 89557, USA; sv.khaiboullina@gmail.com; 4 Institute of Fundamental Medicine and Biology, Kazan Federal University, Kazan, 420008 Tatarstan, Russia

**Keywords:** SARS-CoV2, genome, mutation, bioinformatics

## Abstract

SARS-CoV-2 has spread very quickly from its first reported case on 19 January 2020 in the United Stated of America, leading WHO to declare pandemic by 11 March 2020. RNA viruses accumulate mutations following replication and passage in human population, which prompted us to determine the rate and the regions (hotspots) of the viral genome with high rates of mutation. We analyzed the rate of mutation accumulation over a period of 11 weeks (submitted between 19th January to 15 April 2020) in USA SARS-CoV-2 genome. Our analysis identified that majority of the viral genes accumulated mutations, although with varying rates and these included NSP2, NSP3, RdRp, helicase, Spike, ORF3a, ORF8, and Nucleocapsid protein. Sixteen mutations accumulated in Spike protein in which four mutations are located in the receptor binding domain. Intriguingly, we identified a fair number of viral proteins (NSP7, NSP9, NSP10, NSP11, Envelop, ORF6, and ORF7b proteins), which did not accumulate any mutation. Limited changes in these proteins may suggest that they have conserved functions, which are essential for virus propagation. This provides a basis for a better understanding of the genetic variation in SARS-CoV-2 circulating in the US, which could help in identifying potential therapeutic targets for controlling COVID-19.

## 1. Introduction

The outbreak of a severe viral pneumonia in Wuhan, China, 2019, led to the discovery of the new strain of the coronavirus [1]. Soon, the International Committee on Taxonomy of Viruses proposed the name SARS-CoV-2, while the name coronavirus disease 19 (COVID-19) was adapted by the World Health Organization (WHO) to avoid confusion with the previous SARS outbreak in 2003 [2]. SARS-CoV-2 is a novel member of the Coronaviridae family and belongs to the genus *Betacoronavirus* [3]. Coronaviruses are zoonotic, believed to be originated from bats, and circulating in civets, pangolins, and dromedary camels [4,5,6,7]. Coronaviridae consists of two genera, alphacoronavirus and betacoronavirus, infecting humans and causing respiratory problems. In healthy adults, infection is usually mild, while elderly and patients with comorbid factors experience severe and sometimes fatal consequences [8].

SARS-CoV-2 is an enveloped virus with a positive-sense single strand RNA genome [9]. The viral genome is one of the largest among RNA viruses with a size of 29,903 kb [10]. The viral genome codes for structural and non-structural proteins (nsp). The structural proteins include envelope (E), matrix (M), nucleocapsid (N), and spike (S) that function to protect the viral genome (E, M, and N) which bind to the host cell receptor (S) [11]. Sixteen non-structural proteins regulate coronavirus RNA synthesis and processing, such as RNA dependent RNA polymerase (RdRp) (nsp12), helicase (nsp13), mRNA capping (nsp14 and nsp16), and fidelity control (nsp14) [12]. Several proteins, such as nsp7 and nsp10, a crucial cofactor facilitating the function of viral enzymes, are also coded by the viral genome. Coronavirus evolution employs point mutations and recombination [11]. Mutations could be the result of the RdRp fidelity errors or a directed response to altered selective pressures on the viral genome [13]. Coronaviruses also utilize homologous recombination, where the virus could exchange larger genetic material supported by the data that Bat-SCoV could be the product of a recombination event between the ORF1 and ORF2 [14]. Also, HCoV-NL63 could be the product of multiple recombination events [15]. These mutations could affect virus infectivity, as was recently demonstrated for COVID-19, where the receptor binding domain (RBD) recombination and a cleavage site insertion were suggested to increase the efficacy of virus entry into the host cell [16].

It appears that the mutation rate of SARS-CoV-2 is lower as compared to SARS-CoV [17]. Recent studies showed that the homology of the viral nucleotide and amino acid sequences is high, however they have identified several high mutation frequency regions in the SARS-CoV-2 genome [18]. These regions included ORF1a, S, and N encoding genes to have a high frequency [18,19,20]. Interestingly, mutations in S and RdRp proteins were most commonly found in the European population, where nucleotide changes in these genes appear to be the result of co-evolution [21]. It also was suggested that some mutations, especially in the S protein, could be linked to the virus spread and pathogenicity [22]. Several RBD mutations identified in France were shown to increase the receptor binding capacity, which could contribute to high virus spread and severity of the disease [23]. In contrast, a mutation in the S protein was linked to potentially reduced receptor binding affinity [17,24]. It appears that mutations in some regions of the SARS-CoV-2 genome could be essential for the spread of this pandemic and severity of the disease.

The COVID-19 virus, originated in China, is now diagnosed in more than 181 countries [25]. Interestingly, the number of cases and fatality rate differs from country to country [26]. Although multiple factors could affect the susceptibility of infection and the death rate, virus mutation and adaptation to the new environment could also play an important role. A report supports this assumption on mutation in the S protein of COVID-19 shown to decrease its binding to the receptor [17]. Although changes in the SARS-CoV-2 genome were demonstrated, little is known about the mutation rate in this virus circulating currently. This study analyzed mutational changes in COVID-19 genomic regions as well as in the proteins using SARS-CoV-2 genome sequences from the Unites States of America during 19 Jan till 15 April. Obtained results could assist in developing a model for accessing the mutation rate, which can be used in predicting future alterations in viral genome and protein sequences. Genes with a high rate of mutation should be further analyzed for their role in SARS-CoV-2 evolution and pathogenesis as they might provide important information on therapeutic targets.

## 2. Results

### 2.1. Genome Analysis

A total of 579 complete SARS-CoV-2 genome sequences from the US patients submitted between 19 January and 15 April 2020 were included in the analysis. Using Jal view visualization, redundant sequences were identified which were removed from the analysis. Genome sequences with characters other than A, T, G, and C such as N, R, X, and Y, represent sequencing errors, such as unspecified or unknown nucleotide, unspecified purine nucleotide, and unspecified pyrimidine nucleotide, respectively were also excluded from the analysis. Additionally, sequences with unknown dates of collection were also removed. After considering all the exclusion criteria, 196 unique SARS-CoV-2 genome sequences remained to use for mutational analysis. The complete sequence of 196 genomes is provided in a Supplementary Material S1, while the accession numbers and collection dates of these sequences are summarized in Supplementary Table S2. 

**Figure 1:**
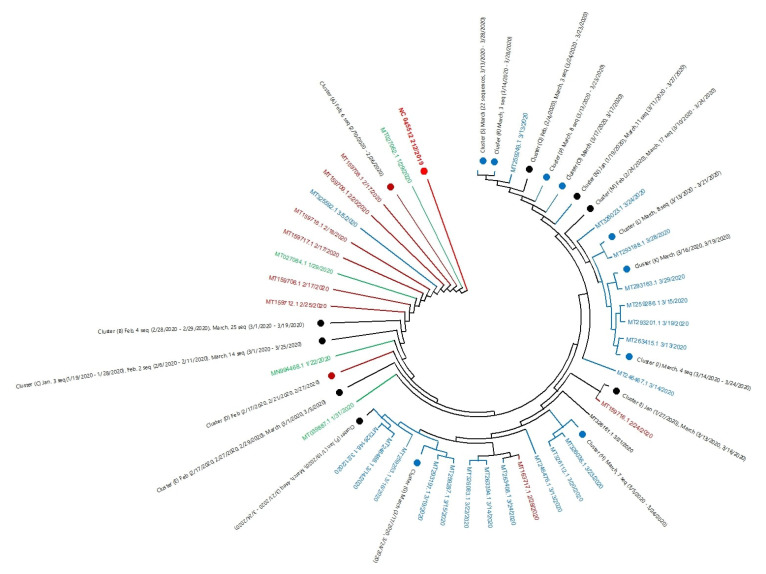
Phylogenetic tree of whole genome 196 sequences of SARS-CoV-2 USA outbreak. The tip of branches corresponds to the accession numbers with released dates of sequences. The taxon colored with green, brown and blue for months January, February and March respectively. Closely related genome sequences with minimum branch deviation (cut off 0.0005) were represented in clusters (cluster A to S), summarized in Supplementary Material Table S2.

The phylogenetic tree of 196 complete SARS-CoV-2 genomic sequences of the USA and reference sequence (NC_045512) of Wuhan, China was constructed according to their collection date using MEGA-X software [27]. The evolutionary analysis was done using the maximum likelihood method and the Tamura–Nei model [28]. The tree reveals the history of the common ancestry of all 196 SARS-CoV-2 genome sequences from USA outbreak (Figure 1). The lines of a tree represent evolutionary lineages with the highest log likelihood (−2,497,132.35). Sequences were grouped by the taxon and shown as green, brown, and blue colors for January, February, and March, respectively. The tree shows the USA SARS-CoV-2 sequences differ from the reference sequence, NC_045512 of Wuhan, China. Also, the USA SARS-CoV-2 lineage is split into many sub-lineages, which are represented by different branches. Each branch of the tree ends with a cluster or a single sequence.

Closely related genome sequences having a minimum branch deviation (cut off 0.0005) were grouped in clusters (cluster A to S), summarized in Supplementary Table S3. The clusters details are provided in Supplementary Table S3. As an example, cluster A is formed by six closely related genome sequences from February 2020 (MT118835.1 (23-2-2020), MT106053.1 (10-02-2020), MT159720.1(21-02-2020), MT159715.1b (24-02-2020), MT159707.1 (17-02-2020), and MT276324.1 (26-02-2020). We also found that some genome sequences, although isolated during the different months (February or March), still closely related and, therefore, grouped in the same cluster (cluster B). The phylogenetic tree revealed that the genome of more closely related sequences has a low evolutionary rate. Also, the evolution pattern suggests that all lineages share the same ancestry, such as the Wuhan virus with multiple gene mutations over time.

Figure 1 also demonstrates that all March sequences were more distant from the sequence of Wuhan except MT325592.1 (3/5/2020). Our findings support the notion that the US SARS-CoV-2 virus genome is the product of the Wuhan SARS-CoV-2 evolution. However, it appears that the virus genome is continuously changing and that could be a result of adaptation to the new environment. 

### 2.2. Mutational Analysis in Genome

**Figure 2:**
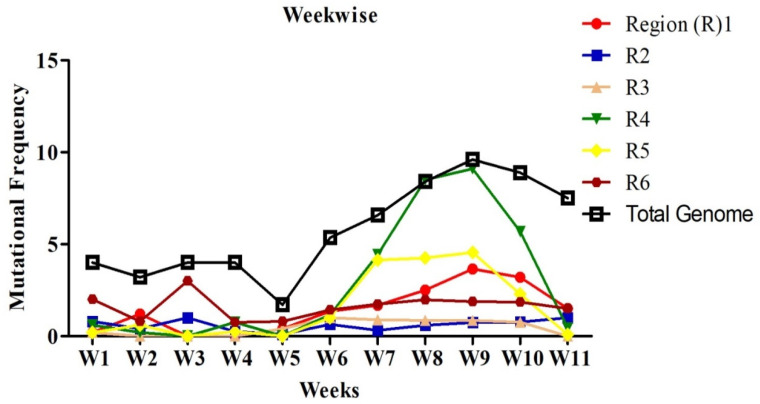
Nucleotide mutational frequency of six genomic segments of SARS-CoV-2. Mutational frequency was calculated by the ratio of the number of total nucleotide mutations and the number of genome sequences in each week. The SARS-CoV-2 genome was divided into six regions, which are represented as R1–R6.

**Figure 3:**
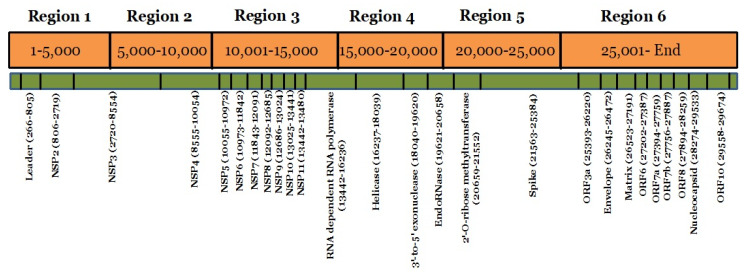
Mapping of SARS-CoV-2 genome regions and proteins. The SARS-CoV-2 genome was divided into six regions and the location of each protein in the different regions is schematically presented.

**Figure 4:**
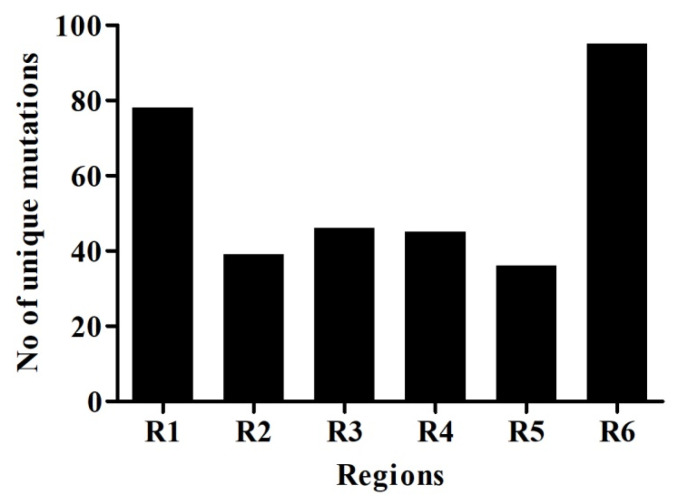
Unique nucleotide mutation of six genomic regions of SARS-CoV-2. Unique mutations are calculated by removing the redundant mutations, which occur in more than one week. The SARS-CoV-2 genome was divided into six regions, which are represented as R1–R6.

Mutations were grouped by the date and divided into seven days period, making a total of 11 weeks. Mutation frequency was calculated by taking the ratio of the number of total nucleotide mutations and the number of genome sequences in each week. Mutation frequency for whole genomes was observed to be low during the initial five weeks. However, after the first five weeks, the frequency seems to have increased sharply until week nine and remained similar or even slightly down in weeks 10 and 11 (Figure 2). To identify the regions with the most mutations over time, SARS-CoV-2 genome was divided into six regions of approximately 5 kb each and these were named as region 1 (1–5000 bp), region 2 (5001–10,000 bp), region 3 (10,001–15,000 bp), region 4 (15,001–20,000 bp), region 5 (20,001–25,000 bp), and region 6 (25,001–end) (Figure 3). This was done to facilitate the analysis of the large genome of the SARS-CoV-2 virus, which is 29,903 kb [10]. It appears that the mutational frequency of regions 1 (1–5000 bp), region 4 (15,001–20,000 bp), and region 5 (20,001–25,000 bp) increased over time with the higher frequency in weeks 7–10. Overall, the mutation frequency during entire period of analysis (11 weeks) was found to be highest in region 4, followed by regions 1, 5, and 6 (Figure 2). Regions 2 and 3 had the lowest mutation frequency and appeared to be more conserved. Unique mutations are also calculated by removing the redundant mutations identified in more than one week. Unique mutations per region per week are summarized in Supplementary Table S4. The total number of unique mutations accumulated during the entire 11 weeks were found to be highest in region 6 followed by region 1 as shown in Figure 4. 

### 2.3. Mutational Analysis of COVID19 Proteins

**Figure 5:**
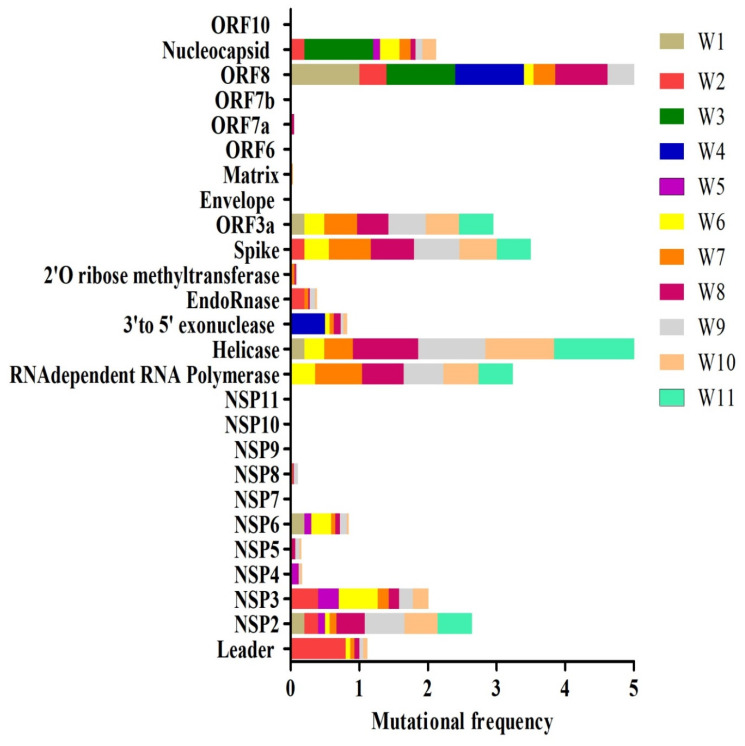
Week wise comparative amino acid mutational frequency of SARS-CoV-2 proteins. Mutational frequency was calculated by the ratio of the number of total amino acid mutations and the number of genome sequences in each week. W1–W11 represents different weeks.

The position of viral proteins was identified using the Swiss model and Genbank. Additionally, the amino acid mutations were attained from the Coronavirus Typing Tool. The amino acid mutations frequencies were calculated and analyzed during a period of 11 weeks for all six regions of SARS-CoV-2. The amino acid mutation frequency for each protein is demonstrated in Figure 5. The proteins such as ORF8 and helicase appear to have the highest mutation rate over the period of 11 weeks (Figure 5). NSP2, NSP3, RdRp (RNA dependent RNA polymerase), S (Spike), ORF3a, and N (Nucleocapsid) also showed substantially high mutation frequency. Interestingly, some proteins such as NSP7, NSP9, NSP10, NSP11, E (Envelope), ORF 6, and ORF 7b had no mutation frequency over the study period. Unique amino acid mutations identified in each week are summarized in Supplementary Table S4. Additionally, the total number of the unique mutations identified in different proteins during the period of 11 weeks study is presented in Table 1. The highest number of unique mutations was found in NSP3 (Table 1). NSP2, RdRp, helicase, S, and N proteins had unique mutations ranging between 12 and 17 amino acids per protein (Table 1). The amino acid position for each protein corresponding to the nucleotide position in the genome is given in Table 1 and Supplementary Table S5. Certain mutations were identified to be sustained in several weeks, for example, T85I (1059C > T), P153L (3177C > T), L37F (11083G > T), P323L (14408C > T), P504L (17747C > T), and D614G (23403A > G), which are sustained in more than five weeks and have been highlighted in red color (Table 1 and Supplementary Table S5). Interestingly, several mutations such as A58T (NSP3), L37F (NSP6), P323L (RdRp), P504L and Y541C (helicase), D614G and H49Y (S), G251V (ORF3a), L84S and V62L (ORF8 protein), R203K, S194L, and S202N and G204R (N) have already been reported previously in different countries [18,29,30,31,32,33]. It should be noted that all these mutations, except A58T (NSP3) and H49Y (surface glycoprotein) were also persistent in different weeks.

### 2.4. Mutational Analysis in Spike Protein

**Figure 6:**
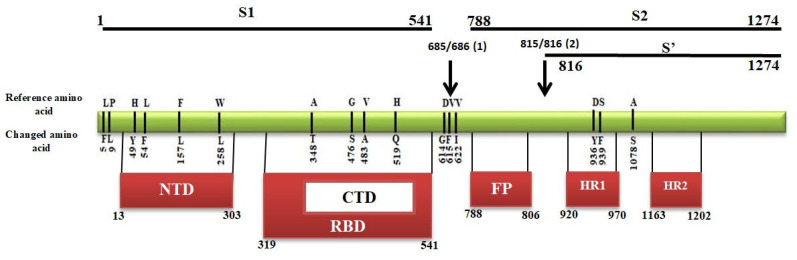
Mapping of mutations in different domain of spike protein. S1 and S2 are subdomains, N-terminal domain (NTD), C-terminal domain (CTD), Receptor binding domain (RBD), Fusion peptides (FP), Heptad repeats (HP), Cleavage sites are represented by the arrow.

Transmembrane S glycoprotein assists the virus entry into the host cell. It is cleaved by the host proteases at two sites (685/686) and (815/816), producing S1, S2, and S’ subunits [34]. The two subunits, distal S1 and membrane-anchored S2, bind to the angiotensin converting enzyme 2 (ACE2) receptor and fuse with the cell membrane, respectively [35]. The S1 subunit contains the N-terminal domain (NTD), C-terminal domain (CTD), and receptor binding domain (RBD). S2 includes the fusion peptide (FP) and heptad repeats (HR1, HR2) regions, while S’ includes heptad repeats (HR1, HR2) regions (Figure 6). All domains are crucial for the virus interaction with the host cells as they function to bind to the receptor and mediate fusion with the host cell membrane [36,37]. Our analysis identified 16 mutation sites in the S protein, and all these mutations were nonsynonymous. Out of these 16 mutations, four were in NTD fragment, i.e., H > Y(49), L > F(54), F > L(157) and W > L(258) (Figure 6). Also, four mutations were found in the RBD region, A > T(348), G > S(476), V > A(483), and H > Q(519). The remaining eight sites were located in different regions within the protein, L > F(5), P > L(9), D > G(614), V > F(615), V > I(622), D > Y(936), S > F(939), and A > S(1078). The fusion peptide region was highly conserved as no mutation sites were found. 

## 3. Discussion

Identified during the outbreak in Wuhan in December 2019, SARS-CoV-2 is currently diagnosed in 181 countries and regions, rendering the first pandemic in 21 century [25,38]. The Wuhan SARS-CoV-2 strain has more than 80% identity with SARS-CoV (originated in bats) and 50% with MERS-CoV (originated in camels).,which originated in bats [39]. It appears that current SARS-CoV-2 is the result of several mutations supporting the notion that virus evolution is an ongoing process; thus, resulting in new strains [40]. The viral genome is translated into 16 nsps coded by two polyproteins, pp1a and pp1ab [9]. The structural proteins, namely S, E, M, and N, are translated from single guided RNAs. Nsp functions to regulate virus replication while structural proteins are involved in binding to the receptor and virion assembly. While switching the host and adapting to a new one, viruses mutate by adjusting to the new environment to ensure better replication. CoVs have already been known to switch hosts (zoonosis) and in this process, they acquire mutations [41]. The receptor binding domain of S protein is known to select specific mutations, which improves its binding to the ACE2 receptor [41]. These mutations enhance the virus entry into the host cell, and therefore virus replication. For instance, some CoV obtained phosphodiesterases, which can cleave and inactivate 2′,5′-oligoadenylate [42], a potent antiviral protein [43] is believed to be developed through convergence and divergence evolution [44].

Often, mutations promoting virus replication are identified at the late stages of the outbreak leading to limited data on the history of the virus evolution and accumulation of the mutations. The current SARS-CoV-2 pandemic provides a unique opportunity to analyze the progression of the virus evolution and identify the mechanisms of selection for favorable mutations. By analyzing 196 complete SARS-CoV-2 genomic sequences from the USA, we were able to track the dynamics of the virus mutations and the viral genome regions most vulnerable to acquire mutations. Our analysis revealed that, although all sub-lineages have the same Wuhan virus ancestry, they did not evolve directly from that virus. Therefore, it could be suggested that viruses from different regions and not China contributed to the SARS-CoV-2 outbreak in the USA.

Mutational frequency of the whole genome has shown a trend of increase over a period of time, which can be associated with an increase in the USA population’s infection rate. To determine the regions with a higher mutation rate, the SARS-CoV-2 genome was divided into six regions, which showed regions 1, 4, 5, and 6 to have a higher mutation rate as compared to regions 2 and 3. The mutational rate was increased over the time when a higher mutational rate was observed in weeks 7–10. Regions 2 and 3 corresponded to NSP4, 5, 6, 7, 8, 9, 10 and 11, which appears to be highly conserved. The conservative nature of these proteins was also confirmed by the results of amino acid mutation analysis where NSP7, 9, 10, and 11 had no mutations. The amino acid mutation frequency was higher in NSP2, NSP3, RdRp, helicase, S, ORF3a, ORF8, and N proteins, but the maximum number of mutations was detected in ORF8 and helicase. Interestingly, all these genes were shown to be under positive selection pressure in SARS-CoV-2 [45,46]. Changes in ORF8 appear to have a strong link to the new species adaptation as substantial alterations were demonstrated in SARS-CoV ORF8 during the switch from civet to human host [7]. Also, the role of ORF8 mutation as a virus adaptation mechanism to human host was shown during the SARS-CoV outbreak in 2003 [47]. Multiple deletions including the large one, containing 415-nt and resulting in the loss of the entire ORF8 region were, described in some patients [48]. Moreover, changes were identified in this ORF between SARS-CoV and SARS-CoV-2 [49], which is now shown to lack the VLVVL motif required for inflammasome activation. Both ORF3 and ORF8 encoded proteins are type I interferon inhibitors [50,51], promoting virus replication by interfering with anti-viral defense. Therefore, it could be suggested that changes in gene coding for N protein as well as ORF3a and ORF8 contribute to virulence, transmission, and pathogenicity during the epidemic [47].

The fourth region of the SARS-CoV-2 genome was the most variable, including the C terminus of RdRp, Helicase, exonuclease, and N terminus of EndoRNAse. Among these genes, Helicase was the most variable. Helicase is essential for viral replication and proliferation [52] as it mediates ATPase as well as DNA and RNA duplex-unwinding activities [53]. Interestingly, a higher number of mutations were also found in RdRp as well. As RdRp physically interacts with Helicase to enhances its unwinding activity [54], it could be suggested that mutations in both proteins at weeks 6–11 are concomitant to ensure the functional compatibility of these proteins.

Another striking observation was the detection of multiple mutations in the Leader protein at an early time in week 2, while a limited number of changes in the genome were detected in the later time points. Leader sequence alteration including deletions and nucleotide substitutions are shown for many coronaviruses [47,55]. These mutations could substantially affect virus replication and were shown to be frequent during the late stage of the epidemic [47,55].

We also identified multiple SARS-CoV-2 genes, which were not mutating during the time of the analysis. These genes code for NSP7, NSP9, NSP10, NSP11, E, ORF 6, and ORF 7b accessory proteins. These proteins have multiple functions including type I IFN production, cleavage of procaspase 3 (ORF7b), promoting virus release, interaction with M protein (E), interacting with NSP8 (NSP7), binding to RNA (NSP9) and acting as a co-factor for NSP10 (NSP9) [56,57]. Similar to our finding, no mutations were found in NSP9, while only two amino acid substitutions were identified in NSP10 [58]. Also, the role of NSP9 and NSP10 in SARS-CoV-2 pathogenesis was demonstrated, where the binding to NFκ-B repressing factor appears to facilitate IL-6 and IL-8 production [58]. These cytokines play a key role in neutrophil infiltration and inflammation [59]. Therefore, it could be suggested that local inflammation is essential for SARS-CoV-2 propagation, as it retains these NSPs unaffected during the study period. Also, NSP9 and NSP10 could be potential targets for the treatment of SARS-CoV-2 to reduce local inflammation and tissue damage.

The mutations may vary in different countries due to the host selection pressure. Our analysis included SARS-CoV-2 virus sequences collected in the USA. Several mutations of SARS-CoV-2 proteins were found to be persistent over the weeks of analysis in the USA. Some of the persistent mutations were reported in studies focused on virus sequences obtained in different countries, suggesting similarities in SARS-CoV-2 evolution across the world [22,23,24,25,26,27]. However, it appears that country and continent specific mutations are accumulated as the SARS-CoV-2 virus evolves. The mutation frequency of thirteen nucleotides in four geographic areas (Asia, Oceania, Europe, North America) was found to differ within these geographic areas [29]. Out of these thirteen mutations, seven, 2891 (A 58 T), 14,408 (P to L), 17,746 (P to L), 17,857 (C to Y), 23,403 (D to G), 28,144 (L to S), and 28,881 (R to K), were also identified in our study. These data suggest that, although some mutations are intrinsic for SARS-CoV-2 evolution, certain mutations could be the result of virus adaptation to the specific environment in a given country. This environment could include the age variation within the population, access to health care and socio-economic factors.

The presence of mutations similar to those identified in other parts of the globe suggests that these mutations facilitate virus adaptation to the human host. These mutations are found in NSP3, NSP6, RdRp, helicase, ORF3a, ORF8, as well as in the S and N proteins. Interestingly, these are the same proteins shown to have the highest mutation rate in our study. These proteins play a role in adsorption, replication and polyprotein processing, which are essential for coronavirus replication. A total of sixteen mutations were found in the S protein located in different domains. The majority of these mutations (thirteen) were found in the S1 subunit, whereas only four are present in the RBD domain. RBD is the crucial domain that is responsible for binding to the ACE2 receptor [60]. Most of the antibody isolates from patient blood are shown to target the S1 subunit, typically RBD domain [61,62]. Therefore, it could be suggested that a high rate of mutations in this domain could affect the antibody binding to this protein, promoting virus infection and facilitating pathogen evasion of the host immune control. S2 and S’ subunits appear to be relatively conserved as only three mutations are found. This low rate of mutation in this subunit could indicate the importance of the structural stability of this protein for virus replication. This region contains FP, which can form an extended bipartite fusion platform capable of penetrating deeper into the membrane, which is essential for host cell infection [36,37,63]. These data suggest that the FP region of S2 could be a potential target for the development of a vaccine and therapeutics. 

Our data demonstrate that SARS-CoV-2 genome accumulated mutations during the first 11 weeks of the outbreak in the US. These mutations could affect virus replication as well as host immune reaction to the pathogen. It appears that the virus extensively utilizes the mechanisms to inhibit host defense while promoting inflammation. It is unclear what the role of local inflammation is in the SARS-CoV-2 pathogenesis, however a limited number of mutations in the genes coding for proteins facilitating inflammatory cytokines and chemokines production suggest that inflammatory milieu is important for virus propagation. 

## 4. Material and Methods

### 4.1. Genome Sequences Retrieval

The genomic sequences allow information on synonymous and nonsynonymous variants within the time that directly affects proteins encoding. The complete genome sequences of SARS-CoV2 collected in the USA outbreak along with the collection date were retrieved from the NCBI virus database (https://www.ncbi.nlm.nih.gov/nuccore/?term=COVID19). Multiple sequence alignment was performed using a virus pathogen resource (https://www.viprbrc.org/) because of the sizeable sequential data set. The genome sequence redundancy was removed through Jalview visualization [64]. 

### 4.2. Genome Analysis

The nucleotide and amino acid position of each protein of SARS-CoV-2 genome was located using Swiss model repository (https://swissmodel.expasy.org/repository/species/2697049) of SARS-COV2 and Genbank. The genome analysis was executed by using a free web-based tool, the Coronavirus Typing Tool (2020) which performs phylogenetic analysis to identify clusters present in diverse sequences of SARS-CoV-2 [65]. It facilitates the identification of coronavirus types including SARS-CoV-2 and genotypes of a nucleotide sequence. Nucleotide sequences in the FASTA format retrieved from NCBI were given as an input in the tool to get mutational information of the questioned genome in reference to the sequence of virus isolated from Wuhan sea food market (NC_045512). Nucleotide and protein mutation examination was accomplished manually using Coronavirus Typing Tool (2020). Mutation frequency for nucleotide and amino acid changes were calculated for each week. The nucleotide and amino acid mutations present in all genomes obtained during a particular week were clubbed to calculate total number of mutations. The ratio of the total number of mutations in each week and total number of genomes obtained in that week was used to calculate the mutation frequency.

### 4.3. Phylogenetic Analysis

Selection of sequences for analysis, alignment of genomic or proteomic sequences, tree building, and tree evaluation are the most critical factors to explain the molecular evolution process of an organism. To get an actual association among examined sequences, the maximum likelihood method was used from MEGA X [27]. In this method, the likelihood is calculated for each nucleotide substitution in the alignment. This is the most computationally intensive but flexible method for determining topology and branch lengths [66]. It renders the statistical model for evolutionary diversity that varies across branches.

## Figures and Tables

**Table 1:** Unique mutations, which are persisted over the week in SARS-CoV-2 protein.

Protein	Unique Mutations
Leader	G82_V86del (509_523delGGTCATGTTATGGTT), K141_F143del (686_694delAAGTCATTT), D75*E (490**T > A), A117T (614G > A), M1L (266A > T), L21S (327T > C) [6 ***]
NSP2	F10L (833T > C), D43N (932G > A), T85I (1059C > T), N98S (1098A > G), P129L (1191C > T), H194Y (1385C > T), G199E (1401G > A), G212D (1440G > A), T223I (1473C > T), S248N (1548G > A), V311M (1736G > A), K337R (1815A > G), G339S (1820G > A), A361V (1887C > T), T429I (2091C > T), V480A (2244T > C), M609I (2632G > T) **[17]**
NSP3	A58T (2891G > A) **, A655V (4683C > T), T127I (3099C > T), P153L (3177C > T), Q180H (3259G > T), T217I (3369C > T), D218E (3373C > A), A231V (3411C > T), P340L (3738C > T), D410Y (3947G > T), K412N (3955G > T), N506S (4236A > G), S697F (4809C > T), S721del (4880_4882delAGT), T763M (5007C > T), P778L (5052C > T), I789V (5084A > G), M951I (5572G > T), T1004I (5730C > T), T1022I (5784C > T), K1042N (5845A > T), S1106G (6035A > G), A1179V (6255C > T), V1209A (6345T > C), T1306I (6636C > T), K1325R (6693A > G), T1482I (7164C > T), N1587S (7479A > G), A1600V (7518C > T), R1614K (7560G > A), K1771R (8031A > G) **[31]**
NSP4	A307V (9474C > T), T327N (9534C > A), A128V (8937C > T), I43V (8681A > G) **[4]**
NSP5	L89F (10319C > T), A173V (10572C > T, T190I (10623C > T), A255V (10818C > T) **[4]**
NSP6	L37F (11083G > T), L260F (11750C > T), V149F (11417G > T), F191del (11543_11545delTTT)I189T, (11538T > C) **[5]**
NSP7	**No mutations**
NSP8	T187I (12651C > T), S41F (12213C > T) **[2]**
NSP9	**No mutations**
NSP10	**No mutations**
NSP11	**No mutations**
RNA dependent RNA polymerase	G44V (13571G > T), K103R (13748A > G), M110V (13768A > G), Q191L (14012A > T), P323L (14408C > T), L372F (14554C > T), K426N (14718G > T), N491S (14912A > G), E744D (15672G > T), G774S (15760G > A), K780T (15779A > C), H810L (15869A > T) [12]
Helicase	A18V (16289C > T), G54C (16396G > T), P77L (16466C > T), P78S (16468C > T), K131R (16628A > G), V226L (16912G > T), T255I (17000C > T), P364S (17326C > T), R392C (17410C > T), T413I (17474C > T), K460R (17615A > G), S468L (17639C > T), P504L (17747C > T), Y541C (17858A > G), T550A (17884A > G), A553V (17894C > T), M576I (17964G > A) **[17]**
3′to 5′ exonuclease	F233L (18736T > C), V287F (18898 G > T), D379A (19175A > C), M501I (19542G > T) **[4]**
EndoRnase	N4K (19632T > A), V9F (19645G > T), V22L (19684G > T), G76D (19847G > A), V127F (19999G > T), F221L (20281T > C), L227del (20299_20301delTTA) **[7]**
2′O Ribose methyltransferase	Y242_S243insF (21384_21385insTTC), S243F (21386C > T), G265V (21452G > T) **[3]**
Spike	L5F (21575C > T), P9L (21588C > T), H49Y (21707C > T), L54F (21724G > C), F157L (22033C > A), W258L (22335G > T), A348T (22604G > A), G476S (22988G > A), V483A (23010T > C), H519Q (23119T > A), D614G (23403A > G), V615F (23405G > T), V622I (23426G > A), D936Y (24368G > T), S939F (24378C > T), A1078S (24794G > T) **[16]**
ORF3a	V13L (25429G > T), L53F (25549C > T), F56C (25559T > G), Q57H (25563G > T), V88A (25655T > C), A99V (25688C > T), T151I (25844C > T), E239D (26109G > T), G251V (26144G > T) **[9]**
Envelope	**No mutations**
Matrix	D3G (26530A > G) **[1]**
ORF6	V9F (27226G > T) **[1]**
ORF 7a	S81L (27635C > T), I110T (27722T > C) **[2]**
ORF7b	**No mutations**
ORF8	M1T (27895T > C), T11A (27924A > G), T11I (27925C > T), S24L (27964C > T), P36S (27999C > T), V62L (28077G > C), S69L (28099C > T), L84S (28144T > C) **[8]**
Nucleocapsid	P6T (28289C > A), S23T (28340T > A), P46S (28409C > T), A152S (28727G > T), S183Y (28821C > A), R185C (28826C > T), S194L (28854C > T), S202N (28878G > A),R203K (28881G > A 28882G > A), G204R (28883G > C), T205I (28887C > T), S232T (28968G > C 28969C > T) **[12]**
ORF10	**No mutations**

* Amino acid position is given as per each protein. ** Nucleotide is represented as per genome position. *** Number indicates total unique mutations. Red highlights are the mutations, which are consistent and occurred in more than one in weeks. Underline Mutations are those, which have been reported, earlier in different literature.

## Supplementary Materials

The following are available online at https://www.mdpi.com/2076-0817/9/7/565/s1, Supplementary Material S1: Retrieved 196 genome sequences of USA outbreak from NCBI virus database. Table S2: Details of 196 sequence of USA outbreak, Table S3: Phylogenetic tree cluster detail of SARS-COV-2 genome of USA outbreak, Table S4: Week wise unique mutations of different region of SARS-COV-2, Table S5: Week wise unique mutations of SARS-COV-2 protein.
